# Recent Progress in Flexible Wearable Sensors for Vital Sign Monitoring

**DOI:** 10.3390/s20144009

**Published:** 2020-07-19

**Authors:** Jihong Liu, Meilin Liu, Yu Bai, Jiahao Zhang, Hongwei Liu, Wenbin Zhu

**Affiliations:** College of Information Science and Engineering, Northeastern University, Shenyang 110819, China; 20173961@stu.neu.edu.cn (M.L.); 20174015@stu.neu.edu.cn (Y.B.); 20173946@stu.neu.edu.cn (J.Z.); 20174188@stu.neu.edu.cn (H.L.); 20173906@stu.neu.edu.cn (W.Z.)

**Keywords:** flexible electronics, wearable devices, biomedical monitoring, health monitoring

## Abstract

With the development of flexible electronic materials, as well as the wide development and application of smartphones, the cloud, and wireless systems, flexible wearable sensor technology has a significant and far-reaching impact on the realization of personalized medical care and the reform of the consumer market in the future. However, due to the high requirements for accuracy, reliability, low power consumption, and less data error, the development of these potential areas is full of challenges. In order to solve these problems, this review mainly searches the literature from 2008 to May 2020, based on the PRISMA process. Based on them, this paper reviews the latest research progress of new flexible materials and different types of sensors for monitoring vital signs (including electrophysiological signals, body temperature, and respiratory frequency) in recent years. These materials and sensors can help realize accurate signal detection based on comfortable and sustainable observation, and may likely be applied to future daily clothing.

## 1. Introduction

In recent years, many new health-tracking devices and smartwatches have appeared in several global electronic product consumer exhibitions. The concept of wearable devices continues to be hot, and the market demand continues to grow. According to the latest data from the International Data Corporation (IDC) [1], global wearable device shipments grew 82.3% in the fourth quarter of 2019 to 118.9 million units. Throughout the year, global wearable device shipments reached 335.5 million in 2019, an increase of 89% compared with 2018. At present, the product forms of wearable devices mainly include smart glasses, smartwatches, smart bracelets, etc. By connecting to the Internet and combining with various kinds of software, we can provide consumers with some relevant vital signs information and keep users in touch in a way that other devices (even smartphones) cannot, especially for improving the current medical disputes between doctors and patients, and effectively configuring the short medical treatment resources play an important role. However, in practical clinical applications, wearable devices should not only ensure the accuracy of signal acquisition but also ensure comfort in the process of wearing as much as possible. By reducing the volume of implantable devices, improving their biocompatibility and endurance, combined with minimally invasive surgery, the invasive injury to the human body should be reduced as much as possible [2], which limits its development. In addition, the comfort experience of consumers is also affected by the stability of battery power supply, service life, sensor placement, power consumption, accuracy, etc.

Sensors are the core of wearable monitoring equipment. In wearable devices (such as wrist strap or chest strap), the sensor will inevitably contact with the skin. This skin contact can cause many problems, such as discomfort due to the presence of sensory nerves and steam on sweaty skin. If a rigid sensor is used, not only will the wearing comfort be affected, but also the signal error of some motion artifacts will be caused. This is because the rigid material does not have enough flexibility and adaptability [3]. Nowadays, wearable devices have low precision, high power consumption and high stiffness, which are mainly due to the characteristics of sensing elements and the need for extensive signal processing to remove noise and extract required features. Therefore, the development of new sensors is of great significance to meet the above challenges. The latest development of nano materials, flexible electronics, and intelligent textiles will bring new development opportunities for wearable devices and practical clinical applications. The new materials used in these sensors are lightweight and natural fitting, thus providing better signal quality.

In this paper, the research progress of wearable health monitoring in materials and sensor technology at home and abroad in recent years is reviewed, and their advantages and disadvantages are highlighted. Section 2 focuses on the materials and sensors of electrophysiological signal monitoring, including the research progress of implantable and non-implantable physiological signal detection of electroencephalogram (EEG), electrocardiograph (ECG), and electromyography (EMG). We also make further investigation and research on the wearable electrophysiological signal sensors which are increasingly popular at present. In Section 3 and Section 4, we describe the research progress in some key areas, such as the respiratory rate and body temperature measurement.

## 2. Bioelectric Signal Monitoring

With the acceleration of people’s social life rhythm, it becomes more and more important to continuously monitor the physiological parameters of the human body. The traditional wet electrode clinical system (i.e., Ag/AgCl electrode) can obtain high-quality signals, but, in the long-term monitoring or wearable applications, the traditional wet electrode has obvious limitations. Because of the need for the participation of conductive paste, the signal quality will be reduced and the wearing comfort will be affected when the conductive paste is dry for a long time. In addition, they may cause skin irritation and allergic contact dermatitis [4]. In order to solve this limitation, there are two solutions, one is a contact electrode without conductive paste, the other is a capacitive non-contact electrode. The flexible electrode is a more reasonable and comfortable solution than the rigid ones. Flexible electrodes are generally divided into two types. One is the self-supporting electrode with excellent electrical conductivity and electrochemical activity at the same time, such as activated carbon, graphene, carbon nanotube flexible electrode, etc., which integrates fluid and active materials. The other is a flexible electrode made by loading electrochemical active material on a flexible fluid collector with high conductivity. Since charge adsorption/desorption only occurs on the electrode surface and the process is completely reversible, the electrode material will not be damaged in the electrochemical reaction process, so the supercapacitor prepared by carbon material has the advantages of high-power density, good performance, and long cycle life. The role of flexible packaging materials is to provide external protection and mechanical support. Thus, on account of the excellent mechanical properties of flexible plastic sealing materials, polyethylene terephthalate (PET) and polydimethylsiloxane (PDMS) are generally chosen. The role of the diaphragm is to prevent the electrode short circuit caused by the contact of the anode and cathode. To make the electrochemical active materials on the positive and negative electrodes fully participate in the reaction, the materials with good wettability to the electrolyte are generally selected. To make the electrochemical active materials on the anode and cathode fully participate in the reaction, the electrolyte is generally selected to have good wettability [5]. Therefore, we have reviewed the research progress of flexible bioelectrical signal sensors in recent years.

In order to improve the accuracy of signal monitoring and diagnosis and user comfort, people have made a long-term exploration and improvement on materials, power consumption, life, and wearing parts for wearable devices. Bioelectric monitoring is susceptible to noise interference, so reducing its motion artifacts, prolonging the effective monitoring time, improving biocompatibility and improving the quality of signal acquisition are the main research focuses. At present, wearable devices are mainly divided into two types: (a) non-implantable recording device, whose sensing electrode is attached to the patient’s body surface (usually the chest or the arm), which is suitable for short-term recording; (b) implantable circulatory recorder. Although subcutaneous implantation can monitor the heart rhythm for a long time, the cost of the device itself and hospitalization is considerable, including surgical risks. Table 1 lists some typical examples of wearable vital signs monitoring that we will discuss later, as well as relevant features and limitations for comparative analysis.

### 2.1. Non-Implantable Flexible Electrophysiological Signal Sensor

#### 2.1.1. Contact Sensors

There are many kinds of non-implantable contact electrodes, including wet electrodes, semi dry electrodes, and dry electrodes. Wet electrodes, as standard electrodes for clinical and research applications, are often made up of Ag/AgCl metals. Conductive adhesives and adhesives are used as contact media for electrodes to reduce contact impedance, which has a good signal-to-noise ratio and reliability. However, due to the presence of hydrogels, skin irritation and discomfort can be caused [24]. For example, the sticking heart monitor patch is easy to use, comfortable to wear and waterproof [25]. However, it is very troublesome to operate, and the quality of the P wave signal is often insufficient. In order to overcome the shortcomings of wet electrode technology, dry electrodes and semi-dry electrodes based on non-conductive paste have become a research hotspot in recent years. Dry electrode has the advantages of binderless, fast assembly and cleaning, but its impedance with skin is low and unstable. There is no metal-electrolyte interface, but there are equivalent circuits (a capacitor is formed between the metal plate cuticle and dermis). An amplifier with a high input impedance is also required. However, capacitance changes as voltage changes and the amplifier with high input impedance is vulnerable to be disturbed, both of which will lead to motion artifacts, and poor signal quality. Compared with the dry electrode, the semi-dry electrode shows certain advantages, but the electrolyte release driven by pressure is easy to cause uncontrollable electrolyte release, thus causing signal instability.

**Figure 1:**
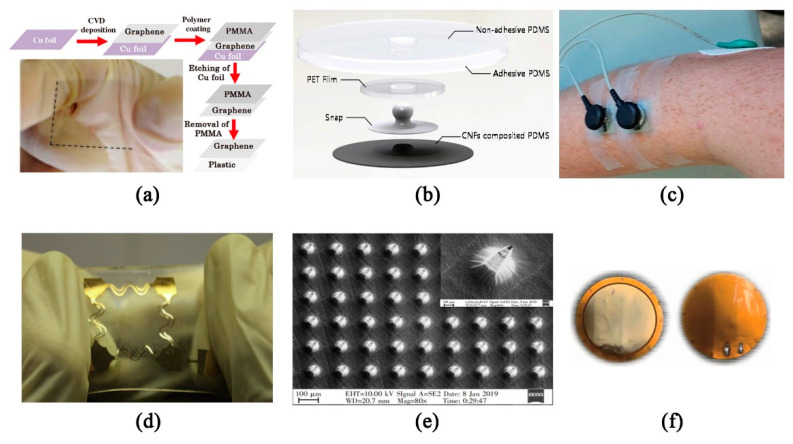
Non-implantable flexible electrophysiological signal sensors: (**a**) schematic diagram of the transfer process and a photograph of the transparent graphene on plastic substrate [29] © 2013 IEEE; (**b**) Schematic image of wearable patches [31] © 2018 IEEE; (**c**) Ag NW electrodes on the forearm for EMG sensing (with black caps) and the ground/reference electrode (with green cap) [6]; (**d**) Camera pictures of electrodes in the case of stretching [3]; (**e**) PDMS flexible micropipette array [37]; (**f**) Pictures of the front and back of the flexible non-contact electrode [15] © 2019 IEEE.

As a good substitute, the flexible dry electrode has made great progress in recent years. Conductive materials mainly include metal, conductive polymer, and graphite [26], etc. For the preparation of electrode materials, the typical methods are as follows. When preparing a metal electrode, Wang et al. [27] proposed a continuous fabrication approach of roll-to-roll ultraviolet-nanoimprint lithography (R2R UV-NIL) for the large-area fabrication of embedded Ag mesh electrodes on polyethylene terephthalate (PET) substrates using the R2R UV-NIL process. A prototype of embedded metal mesh has been fabricated using water-based, nano silver paste, and the electrical and optical performance has been further improved by wet etching. In [28], Ruoff et al. demonstrated the carbon-based supercapacitors fabricated by activation of graphene. The supercapacitors store electrical charge on high-surface-area conducting materials. Their widespread use is limited by their low energy storage density and relatively high effective series resistance. Using the chemical activation of exfoliated graphite oxide, the authors synthesized a porous carbon that yielded high values of gravimetric capacitance and energy density with organic and ionic liquid electrolytes. When making a flexible transparent electrode, graphene synthesized by chemical exfoliation and the CVD process has been recognized as being the most suitable for fabrication. Since the former has many defects, Kalita et al. [29] demonstrate the synthesis of large-area graphene on metal (Ni and Cu) foil by the thermal chemical vapor deposition (CVD) process using the solid camphor (C_10_H_16_O) as a carbon source. The graphene growth process on a polycrystalline metal foil significantly influences by the gas composition and quantity of solid precursor. The synthesis of high-quality continuous graphene film is achieved in the developed technique. A fully flexible transparent conductor is fabricated by transferring the graphene film on a plastic substrate. In the etching process, synthesized graphene film on metal foil was transferred to a PET substrate using a pre-coated Polymethyl methacrylate (PMMA) layer, such that large area thin graphene film does not collapse when transferring (Figure 1a).

When making nanocarbon electrodes, a flexible elastic electrode by using silicone rubber with special carbon black material was successfully fabricated in [30], with an expansion ability of 100% or more. Compared with electrolytepaste electrodes and metal electrodes, it is more suitable for the requirement of comfort in strenuous exercise. Besides, Jung et al. [31] fabricated a novel patch type flexible dry electrode for long-term bio-signal monitoring by mixing carbon nanofibers (CNFs) in biocompatible-elastomer (MED6015) (Figure 1b). The CNFs are high in conductivity, low in price, and are easily dispersed in elastomer uniformly related to CNTs (carbon nanotubes). The fabricated CNF electrodes are coated with sticky elastomer (MG79850) to attach on skin without any other adhesive. Usually, the graphene nanocomposite hydrogel, prepared from graphene oxide (GO), polyvinyl alcohol (PVA), and polydopamine (PDA), exhibits excellent mechanical and electrical properties with a tensile stress of 146.5 KPa, a fracture strain of 2580%. Due to the existence of non-covalent interaction forces such as hydrogen bonding and π-π interaction, it possesses good self-healing with the electrical self-healing efficiency of 98% of its original resistance within 10 s. Due to the maintained catechol groups in the PDA for firm adhesiveness, it shows strong self-adhesion, which helps it identify human activities effectively. The self-healing is an important index for the durability of wearable sensors [32].

For contact electrodes, people have an increasingly tendency to choose fabric electrode and conductive polymer electrode. They are usually made of flexible conductive materials, or non-conductive flexible materials as substrate materials, which are coated with conductive materials. For example, Myers et al. [6] worked on wearable dry electrodes made of silver nanowires (Ag NWs), which are embedded under the surface of an elastic substrate made of polydimethylsiloxane (PDMS) (Figure 1c). Under this static condition, the signal quality of the Ag/AgCl dry electrode is equal to that of the Ag/AgCl wet electrode. It has good antibacterial performance due to the characteristics of silver ion. The same team that used Ag NWs to prepare sensors, such as Zhang, etc. [7], prepared polymer/Ag NW composites by combining a small amount of Ag NWs with a stretchable conductive polymer material with tensile properties up to 500%. Even under 1% strain response, it can also respond to the strain signal in real time. It is verified that the electrocardiographic signal of polymer/Ag NW composite electrodes are comparable with that of commercial Ag/AgCl electrodes, and the effects of signal acquisition are better. These characteristics enable it to be used for omnidirectional sensing and health monitoring of human motion. Beside ECG monitoring, it can also be used for motion, pulse, and vocal cord vibration of human joints. Lam et al. [8] prepared a cotton fabric ECG monitoring flexible dry electrode with cotton fabric as a substrate material and graphene ink as a conductive material. Cotton fabric has the characteristics of flexibility, easy availability, and portability. Compared with the previous research, the development of conductive nanofiber mesh dry electrode, carbon nanotube (CNT) dispersed on PDMS substrate to prepare polymer dry electrode and the development of flexible polymer dry electrode by bonding the micro processed metal sheet to PDMS infrastructure, the manufacturing process is simpler and does not need precise equipment. Based on a kind of biocompatible electro-conductive polymer- poly (3,4-ethylenedioxythiophene): poly (styrene sulfonate) (PEDOT: PSS) loaded laser-induced graphene (LIG), Zahed and others [9] developed a new flexible dry electrode for ECG monitoring. In the process of fabrication, PEDOT: PSS is coated on the direct laser patterned LIG, which has 4-month long-term stability and clear waveforms in all wavebands. This method ensures the comfort of wearing and has good signal quality in a static state, but it is not suitable for dynamic monitoring. Among other studies, the team of Shahandashti [3] proposed a dry flexible electrode, which provides a novel representation for flexible contact electrodes. They chose PDMS as the main material of dry-type electrodes. For the conductive part of electrodes, they chose copper with low resistivity. At the same time, in order to solve the problem of the inflexibility of metal materials, they embedded copper in PDMS substrate in the form of thin film, and shaped the shape of the thin-film metal layer into the form of bending similar to a spring to achieve the scalability of the metal layer, so as to ensure the flexibility and scalability of the entire electrode. Finally, the electrode is tested, and the signal quality is equivalent to the standard wet Ag/AgCl electrode. However, due to the retractable nature of the electrode, the tested object can still fit well with the target when it is in motion and is less sensitive to motion artifacts (Figure 1d), which can be better applied to the monitoring of electrophysiological signals in the scene of target activity.

In addition to the above contact electrodes that are only in contact with the human body surface, there is also an invasive electrode that slightly invades the human body (only reaching the germinal layer of the epidermis). This type of electrode is mainly based on the Micro-Electro-Mechanical System (MEMS) manufacturing process. MEMS manufacturing is based on the development of semiconductor manufacturing technology; it integrates diffusion, thin films (PVD/CVD), photolithography, etching (dry/wet) and other processes as the first stage of the manufacturing process, followed by thinning, cutting, packaging and testing as the second stage, supplemented by precision testing instruments to strictly control the process requirements, to achieve its design requirements. With the development of MEMS preparation technology, in recent years, some scholars have also used MEMS technology to prepare flexible body surface micro-invasive electrophysiological sensors. Moreover, the micro-invasive flexible dry electrode structures prepared by MEMS technology also tend to be diversified, such as columnar [33], planar [34], hemispherical [35], microneedle [36] and other structures. Compared with the dry electrodes of other structures, the micro needle-like dry electrodes need to penetrate into the skin epidermis, only penetrate the cuticle on the upper layer of the epidermis without pain, which is conducive to reduce the interface impedance between the skin and the electrode, and even exceeds the effect of the wet electrode. For example, Bai’s team [37] made the flexible silicon microneedle array dry electrode through the process of photoetching, etching, PDMS morphology transfer in MEMS technology, which not only has good biological compatibility, good flexibility, high stability but also has the advantages of simple process, mass production, and integration. After 3 hours’ attachment, the electrode was better than the traditional Ag/AgCl wet electrode. The transfer morphology of a 180 μm microneedle array after PDMS secondary transfer (Figure 1e) is basically the same as that of a silicon tip. It shows that PDMS not only transfers the tip array structure completely, but also enhances the adhesion between PDMS and metal layer.

#### 2.1.2. Non-Contact Sensors

The above schemes are all contact measurement schemes of a human-body electrical signal. In addition to the measurement by contact method, the electrode cannot directly contact with the skin, and the electrical signal of the human body surface can be extracted by the way of capacitance coupling through clothing. ECG, EMG, ECG, etc., are all internal signals generated by the human body. In the non-contact measurement process, the measuring electrode is placed on the outside of the clothing to obtain the electrical signal of the human body surface. Due to the low frequency of bioelectric signal, the contact impedance between human body surface and electrode can be simply equivalent to coupling capacitance. Since the static electricity on the clothes will also be coupled to the capacitance, the bias resistor R_bias_ should be set at the input end to release the static electricity and reduce the interference [38]. The electrode using this measurement principle is mainly made of hard printed circuit board (PCB). Due to the large impedance of the coupling part, the signal conditioning circuit is required strictly. So we usually use electronic components such as operational amplifier covered on the back as the signal conditioning circuit [4]. For example, Li et al. [13] proposed a wearable non-contact button system based on a non-contact electrode, which uses an ultra-high impedance operational amplifier as the front input at the back of the electrode plate. Then, there is a 0.07-Hz high pass filter, a 60-Hz notch filter and a 100-Hz low pass filter, which can obtain a variety of bioelectric potential signals through multi-layer cloth, including ECG, EMG and EEG, which can be used for long-term bioelectric potential signal monitoring. However, for the scheme of using rigid PCB as an electrode with a signal conditioning circuit, the rigid PCB cannot fit perfectly with the body surface, and the gap between the PCB and the body will change greatly in the motion state, which will cause serious motion artifacts [39].

To solve this problem, the team of Liu et al. [14] made a flexible printed circuit (FPC) flexible circular electrode with a diameter of 2.5 cm, without components on both sides. The flexible non-contact electrode is connected with the main PCB board by using the soft shielded cable. The bioelectric signal carried by the shielded cable is buffered, amplified and digitized on the main PCB and then transmitted to the personal computer via Wi-Fi network. LMP7702 with very high input impedance, high CMRR 130 dB, very low bias current ±200 fA and differential input ADS1299 is selected as the signal conditioning circuit operational amplifier of the main PCB to measure the ECG signal. When the right leg drive circuit is connected to the human body with it, the signal quality is better compared with the wet electrode and there is no obvious power frequency noise, but with high-frequency noise the Q wave is not visible. After that, this team [15] continued to use the flexible non-contact electrode (Figure 1f), which has no components on both sides, to collect and verify the ECG and EEG respectively. Only the follower and the differential amplifier got the acquisition signal. When the canvas is used as the dielectric, the ECG signal has no obvious distortion compared with the wet electrode; when the nylon is used as the dielectric, the P wave has obvious distortion, while the two media have obvious burr and the Q wave is not visible. The α wave of EEG signal can be seen with baseline drift and high frequency noise. As for the EMG, the surface electrodes must satisfy the following three requirements: high mechanical flexibility, high electrode density and high signal integrity. To achieve high electrode density and high signal integrity, a distributed and shared amplifier (DSA) architecture is proposed by [40], which enables an in-situ amplification of the myoelectric signal with a fourfold increase in EMG electrode density. In the latest research, Gao et al. [16] fabricated a flexible capacitive electrode with a flexible signal conditioning circuit on a polyimide substrate, using Boise Idaho FleX™ ASOPA4002, the customized flexible operational amplifier from American Semiconductor company, whose CMOS devices are ultra-thin (less than 40 µm) and completely flexible. Through the electrical performance test, the response to the known analog ECG signal and the human body test, it is confirmed that the signal conditioning circuit made of the operational amplifier made of the flexible device is equivalent to the traditional rigid device.

### 2.2. Implantable Flexible Electrophysiological Signal Sensor

**Figure 2:**
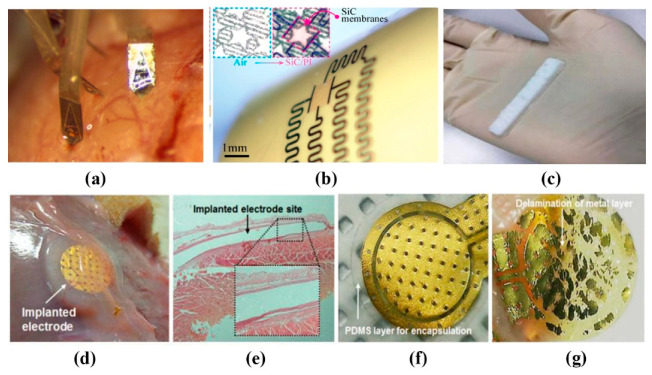
Implantable flexible sensors: (**a**) four independent probes implanted into the mouse visual cortex [46] © 2014 IEEE; (**b**) flexible SiC-on-PI devices wrapped around a curved surface (diameter = 12 mm) [48]; (**c**) the electrode sample prepared in this work. Dimensions (L = 5 cm, W = 0.6 cm and t = 0.2 cm) [21] © 2018 IEEE; photos of implants used in (**d**) biocompatibility tests; (**e**) photos of implants used in biocompatibility tests; (**f**) polydimethylsiloxane (PDMS) encapsulated and (**g**) non encapsulated electrodes [22].

For some dangerous intermittent diseases, such as epilepsy and atrial fibrillation, sustained high-quality ECG signals are needed for health monitoring and auxiliary treatment [41]. And complex brain-computer interface systems, such as neural prosthesis, also need sustained high-quality ECoG signals [42], which requires a biocompatible implantable electrode. The impedance of electrode-tissue interface is one of the main challenges in the design of implantable electrodes. Amani’s team [21] synthesized flexible polymer electrodes from titanium dioxide and organosilicon (Figure 2c). The impedance at 1 kHz is 24 K ohm, which is equivalent to the Cr-Ag-Cr electrode made by Dong et al. [43] and the gold electrode made by Lu et al. [44], but it has better biocompatibility. The team of Lee et al. [22] also used the flexible and implantable capacitance electrode based on the principle of capacitance coupling mentioned above, using the design of polyimide (PI) and Gold/Titanium (Au/Ti) and completely encapsulated it in a PDMS substrate (Figure 2d–g). Due to the inherent characteristics of capacitance coupling, it has obtained a stable and robust ECG signal in the current range of 0–10 mA compared with the direct contact electrode, with almost no leakage current. Wu et al. [45] had made the multifunctional sensors. The conductive composites, hydroxyethyl cellulose (HEC)/soy protein isolate (SPI)/polyaniline (PANI) sponges (HSPSs), were prepared by the lyophilization of HEC/SPI solution and then in situ polymerization of aniline. They can achieve a response time of 0.14 s and efficiently work in vivo for 4 weeks to measure the stimuli, without severe inflammatory reaction. When implanted into different parts of the human body, sensors could detect a variety of electrophysiological signals. Polyaniline (PANI) is chosen as a conductive material, which is a soft and flexible polymer with high electrical conductivity and good adhesive properties. These results show that the electrode can detect a variety of signals stably in vivo for a long time.

Sometimes high-density collection is needed to ensure the measurement accuracy. M. Chamanzar et al. [46] have demonstrated a scalable process for fabricating compact, high-density optrodes for electrophysiology recording and optical stimulation in multiple brain areas. The headstage stack is assembled and fixed to the skull and each probe is independently implanted into the cortex using a robot-assisted stereotaxic micromanipulator (Neurostar, Germany) equipped with piezo-actuated micro-tweezers (Smarac, Germany) (Figure 2a), which allows probes to be inserted into the brain without exerting lateral force that may damage the brain tissue. Besides, using Fabry–Pérot interferometer (FPI) sensors and photonic crystal microcavity structures (PC) micro cavity structures with a free space detection function, as well as two optical communication approaches based on single mode-to-PLGA composite fibers and free-space detection setups, can detect a variety of physiological signals.

Since the existing implantable components are generally permanent and non-absorbable materials, they must be taken out by surgery after use. Shin et al. [47] introduced the absorbable optical sensor implants, which can accurately monitor various internal pressures and temperatures within the clinically relevant time range, and disappear naturally through the biosorption process, eliminating the cost and risk of surgery. Phan et al. [48] present a thin, flexible semiconducting material system that offers attractive attributes. The material consists of crystalline cubic silicon carbide nanomembranes grown on silicon wafers, released and then physically transferred to a final device substrate (e.g., polyimide) (Figure 2b). It has long-term chemical stability in bio-fluids, outstanding water barrier characteristics, and extremely low permeability to ions, which make it an outstanding material for implantable electronics.

For implantable ECG monitoring equipment, the electrode should adhere to the body stably under the condition of movement. Because of the direct contact between the implanted sensor and human tissues, its size and biocompatibility with human tissues are very important. Although most electrode materials are biocompatible, they should be isolated from the tissue to avoid the mechanical stratification of the device itself causing additional stimulation, allergic reaction or inflammation [22]. Implantable cardiac monitors (ICMS) are an excellent tool for assessing rare or potential asymptomatic arrhythmias, and are more often used to monitor atrial fibrillation (AF) [49]. Lee et al. [23] developed an implantable ECG sensor for the real-time monitoring of patients with AF. The Ag/AgCl electrode, the near-field wireless power transmission system and the elastic sealing packaging of polymer film are used to minimize noise, make it biocompatible and maintain air tightness. It is proposed by I. Williams et al. [50] to use silicone as a packaging material of implantable device. They are soft and flexible, which can encapsulate the whole flexible PCB and minimize mechanical damage to surrounding tissue. Considering their relatively high viscosity, people can release potential voids by carefully degassing the MED-6215 silicone and applying the silicone before curing it at a normal pressure, which could otherwise lead to liquid water condensing or even encapsulation failure. Besides, Laiwalla’s team [51] considered that a microscale (500 µm) programmable neural stimulator are hermetically encapsulated using liquid-crystal polymer (LCP) thermocompression for chronic implantability. In fact, a fundamental requirement for the successful operation of implantable systems is an adequate and reliable power supply. Recent research [52] in nanoscale materials for energy generation has created intriguing possibilities for next-generation implantable power sources in the form of flexible and biodegradable batteries and super-capacitors.

### 2.3. Wearable Electrophysiological Monitoring System

#### 2.3.1. Contact Wearable Monitoring System

Embedding sensors and electronic devices in textiles is a trend of wearable devices. The metal electrodes widely used in biomedical signals include silver, copper, aluminum, zinc and gold. Because of their poor performance, corrosion and degradation when exposed to moisture, sweat and washing, the use of textile-based, metal-free electrodes has become a popular choice. It is known that ECG electrodes based on textiles are prone to generate motion artifacts. In order to achieve the stable and convenient detection of physiological electrical signals, many teams are constantly seeking to develop a system to achieve the best results in the use of specific materials and specific wearing parts.

**Figure 3:**
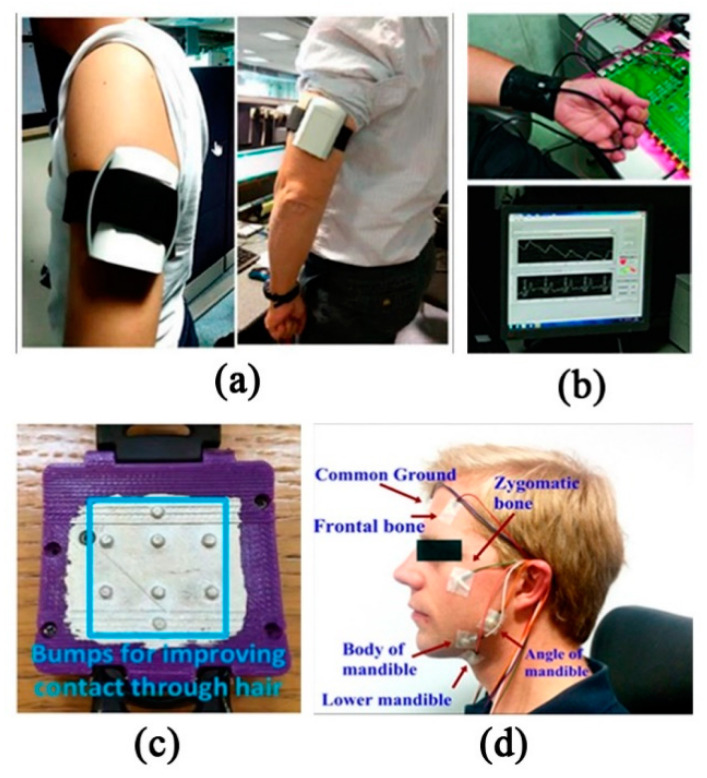
Contact wearable monitoring sensors: (**a**) Arm-mounted electrocardiograph (ECG) acquisition equipment [10]; (**b**) data transmission between infrared PPG transmission belt and USB and a comparison of PPG/ECG signals in the multiple access system [55]; (**c**) bottom view of the case of the electrode in contact with the wrist [11] © 2018 IEEE; and (**d**) the electrodes were placed at eleven positions on the head and neck. [58] © 2016 IEEE.

To explore the function of fabric, Agua et al. [53] compared PEDOT:PSS/divinyl sulfone (DVS) free-standing films with a PEDOT:PSS/DVS-coated textile through experiments. The result turns out that free-standing films can be used in wearable devices attached to the body as a second skin, while textile electrodes are better suited for long-term applications. By introducing the DVS crosslinker, the PEDOT electrodes show remarkable stability in water, without reducing its conductivity. It is also proved that knitted polyester electrodes have the capability to better tolerate friction or skin movements by reducing motion artefacts, and the presence of low-frequency baseline noise can be reduced by means of introducing ionic gels to promote better electrode/skin contact. The wearable arm band ECG acquisition system based on the dry electrode pair of Ag/AgCl by Villegas et al. [10] transmits data to the third-party intelligent device through Wi-Fi for visualization, which greatly improves user comfort. The wireless sensor system can collect, store and visualize the ECG signals recorded from the bipolar wire of the left upper arm (Figure 3a). Limited by battery size and power consumption, the system can only work for 72 h continuously. Vizcaya et al. [54] evaluated the feasibility of interpreting rhythm from far-field bipolar ECG arm band lead recordings on the left upper arm (LUA) in a clinical multichannel arm ECG mapping database (n = 153 subjects). Vinciguerra et al. [55] proposed a low-power portable photoplethysmography (PPG)/ECG combined system (Figure 3b). However, it has poor portability. Users need to connect with computers through a USB port.

Compared with metal-based electrodes, graphene is considered as an alternative and new material for wearable electronic textiles because of its higher flexibility and permeability. Akter Shathi et al. [56] have developed a highly flexible and wearable electronic textile, which is used in the biomedical application of intelligent clothing, especially ECG. Through one bath dyeing and reduction of graphene oxide (GO) with an exhaust dyeing method, it has the characteristics of low impedance and close contact with human skin. By collecting ECG signals of left and right wrists, the fabric electrode can detect a high-quality ECG signal well under wet and dry conditions. Another wearable device proposed by Celik et al. [57] uses the graphene (GN)-coated electrode, which is connected to the back of the ear, upper neck and left arm, and is connected to the measurement module placed on the arm. After the measurement circuit is digitized, amplified and filtered, they specially used Bluetooth to transmit the ECG data to the smartphone for continuous monitoring. Similarly, Beach et al. [11] also proposed wrist band ECG sensor, which integrates ultra-low-power electronic equipment and a personalized 3D printing shell. The convex surface of the shell is to improve the contact with the skin (Figure 3c). In fact, if the electrode is placed on one side of the heart, the amplitude of the collected signal will decrease, and the farther away from the heart, the smaller the signal. Therefore, both hands must be contacted with it to ensure real signal-to-noise ratio (SNR) can be collected. In addition, for dangerous activities such as bicycles, racing cars or military engagements, Wilhelm et al. [58] studied a helmet with embedded sensors, providing the greatest convenience for athletes to monitor their physical conditions at any time (Figure 3d). However, the movement of the head, blinking, jaw clenching and swallowing can cause complex motion artifacts in the recorded ECG; the user friendliness is not so well, and the cable and breathing belt affect the comfort. For people with poor driving technology or driving conditions, the intelligent vehicle controlled by EEG will be a good choice [59]. Its off-line control accuracy can reach 96%, operation control accuracy can reach 95%, and the traffic realization is relatively more convenient and safer.

Another popular wearable monitoring signal is EEG. EEG signals can be used to identify subtle changes in alertness, attention, perception and workload of users in various environments [60]. At present, EEG signals are usually recorded by wet electrodes in hospitals. Although a wet electrode has very low skin electrode contact impedance and good signal quality, the complexity of application and the difficulty of clean conductive gel make it unsuitable for wearable devices. Some studies have suggested that the EEG electrode [61] is made of silver-coated conductive fabric, and it can be used to detect signal without conductive gel on the head. However, the conventional multi-channel work needs wire connection, so it suffers from huge environmental interference, which makes the daily EEG monitoring equipment difficult to be wearable and durable. In order to get rid of circuit constraints and reduce common mode interference as much as possible, some works [62] have designed a kind of wireless active concentric electrode based on body channel communication (BCC): EEG Dust, which is the first distributed active front end (AFE) system with concurrent recording/transmission function so far. In order to get rid of the limitations of gel electrodes and to further monitor the measurement process, Kosmyna’s team [12] introduced AttentivU, a signal detection device similar to glasses. It can simultaneously use EEG and EOG to monitor physiological data in real time. The feedback signal is provided in real time in the form of auditory signal through a bone conduction loudspeaker, without any other equipment.

**Figure 4:**
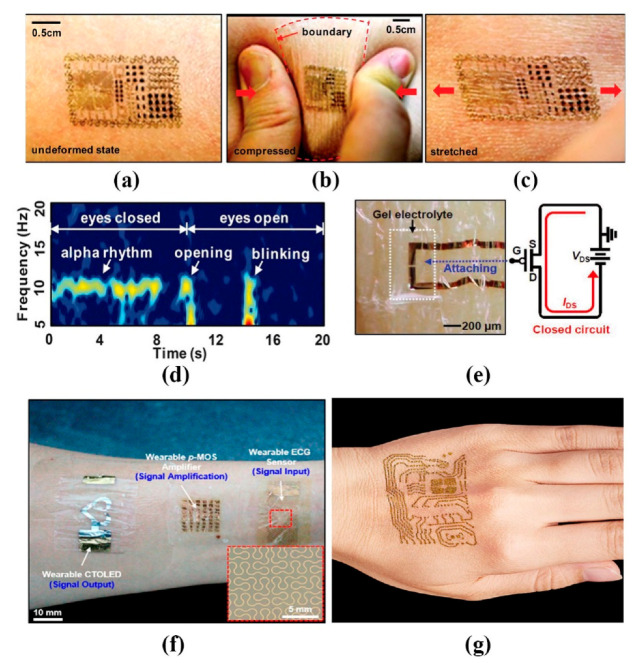
Wearable lightweight sensors: (**a**–**c**) multifunctional epidermal electronic system (EES) on skin: undeformed (**a**), compressed (**b**), and stretched (**c**); (**d**) the spectrogram of the alpha rhythm. The first and next 10 s correspond to periods when the eyes were closed and open, respectively. The responses at ~10 and ~14 s correspond to eye opening and blinking, respectively [63]; (**e**) optical image (left) of the organic electrochemical transistor (OECT)-based sensor on the skin and schematic of its circuit layout (right) [65]; (**f**) photograph of the wearable system for real-time visual monitoring of the ECG signals [66]; (**g**) photograph of ultrathin, gas-permeable nanomesh electrodes fitted with the skin [67] © 2020 IEEE.

As wearables have become more common, sensors have become more sophisticated. Kim et al. [63] made all stuffs integrated on the surface of a thin (~30 mm), gas-permeable elastomeric sheet based on a modified silicone (Smooth-on, Easton, USA) with low Young’s modulus. The devices and interconnects exploit ultrathin layouts designs and the active elements such as silicon and gallium arsenide, are filamentary serpentine nanoribbons and micro- and nano-membranes. It can be attached to the backside of commercial temporary transfer tattoo and adhere robustly to the skin using via van der Waals forces alone. The skin deforms freely and reversibly, without any apparent constraints in motion due to the devices (Figure 4a–c). This epidermal electronic system (EES) can also be applied to other forms of signal detection, such as eye movement, when the EES is mounted onto the forehead (Figure 4d). For long-term use, materials and device strategies to accommodate the continuous efflux of dead cells from the surface of the skin and the processes of transpiration will be needed. But some scholars have suggested that by spraying a layer of adhesive bandage on top of the electrodes after it is adhered to the skin, we can improve the lifetime of the sensor up to about two weeks. This is because the spray-on-bandage material forms a hydrophobic, water-proof surface and also promotes adhesion [64]. This is certainly an important finding. Another considerable find is a new organic electrochemical transistor (OECT)-based electrophysiological sensor that can measure biometric signals with a high SNR (24 decibels) over a long period and even under dry conditions, beyond the limit of the existing OECTs that can only be used temporary when the external solution is used. This is proposed by Lee et al. in [65]. To ensure stable operation, the glycerol was added as a stable medium of the electrolyte in the gel. When the system is working, the changes in the potential of the skin worked as an effective gate bias of the OECT (Figure 1b, right). The gate bias controlled the injection of ions in the gel to the OECT channel and the altered doping state of the channel led to a change in conductivity (Figure 4e). Motion artifacts can be greatly reduced.

Other studies also include considering to show the direct ECG signals’ status directly on the sensors or beside it, which will be more convenient to know about their own heart anytime and anywhere. Koo et al. [66] proposed a wearable electrocardiogram (ECG) monitor based on an ultrathin electrode made of serpentine-shaped thin Au film and a p-MOS CNT signal amplifier, integrated with an ultrathin voltage-dependent color-tunable organic light-emitting diode (CTOLED) for the colorimetric display of the retrieved ECG signals, which involves the use of an ultrathin exciton-blocking layer (EBL) between two emitting layers (EMLs) for blue and red colors (Figure 4f). By optimizing the material and thickness of the EBL, the wearable OLEDs exhibit ECG dependent color changes from dark red, to pale red, to white, to sky blue, and finally to deep blue. It is crucial that the devices perform robustly even after thousands of repetitive deformations and that they withstand consequent fatigues.

To increase the comfortability, Takao Someya et al. [67] proposed so light a sensor that users forget they have it on (Figure 4g). The elastic electrode is constructed of a breathable nanoscale mesh. Nanomesh electrodes are fabricated by electrospinning (a technique in which a strong electric field is applied to the tip of a nozzle to emit a fiber). Unlike the silicone and parylene, nanomesh had the least discomfort. First, nanofibers are formed from polyvinyl alcohol (PVA), then deposit gold on the PVA nanofibers. When it is placed on the skin and sprayed with water, the PVA, which is water-soluble, dissolves and is washed away; only the nanomesh gold wiring remains, which is gas permeable. They do not block sweat glands. In the future, various types, such as smart apparel, band-aid type, and sensors integrated with the skin, will be available depending on the application.

#### 2.3.2. Non-Contact Wearable Monitoring System

**Figure 5:**
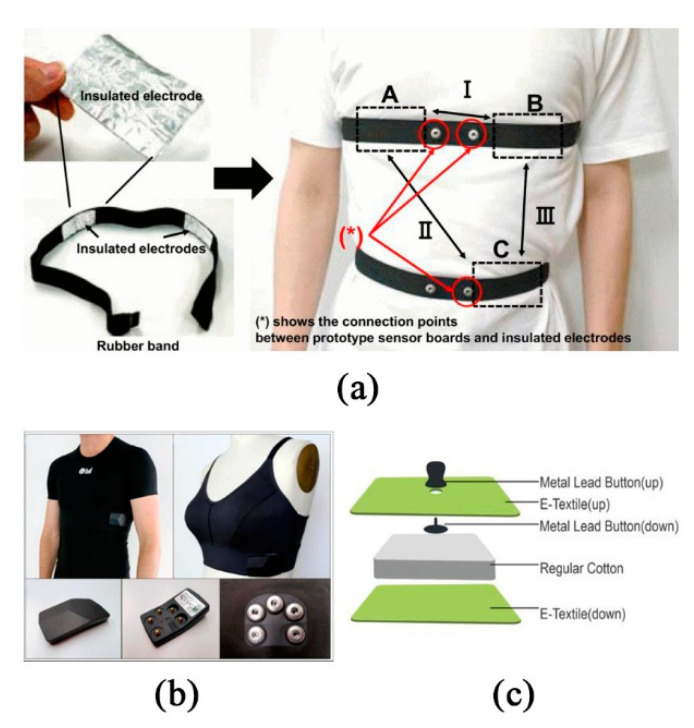
Non-contact sensors: (**a**) insulation electrode and experimental device; (**b**) two different products of OM garments (the OM shirt for men and the OM bra for women [17]); (**c**) the design of sandwich cushion improves the motion artifact [19] © 2020 IEEE.

Compared with the contact ECG measurement device, the emergence of capacitive sensors solves the problem that contact sensors cannot solve in different environments. For example, ECG measurement cannot be easily carried out while driving. In order to prevent the possible emergency caused by the driver’s heart attack or sleeping, the cECG sensor can be installed in the driver’s seat to obtain ECG data [68]. However, the ECG sensor is sensitive to the noise caused by motion [69], so the main problem is to solve the noise pollution. Nagasato et al. [70] proposed a digital auxiliary noise cancellation method for the capacitive coupling ECG sensor, which can improve the availability of the capacitance-coupled ECG sensor, and the maximum power line noise can be suppressed to −29.2 dB. The electrode is made of aluminum foil, fixed inside the rubber band, and placed in A, B, and C positions. When using two-electrode configurations, they get signals by measuring the induction between AB, AC, and BC (Figure 5a). Yapici et al. [20] successfully demonstrated the ECG signal acquisition, processing, and wireless transmission function based on an elastic band by using the superior performance of graphene-coated conductive textiles. Using an elastic band can greatly compensate for the sensitivity of motion artifacts, maintain stable electrode-skin contact, and reduce the contact impedance between the electrode and skin by reducing the air gap between the fabric electrode and the electrode. An adaptive filtering algorithm is used to alleviate the signal distortion caused by wrist torsion. The system can wear ECG monitoring on the wrist or neck with fewer accessories, and avoid the disadvantages of traditional wet electrodes in a long-term monitoring application.

In order to improve the wearability of the flexible electrode, Christian et al. [17] proposed the wearable ECG sensor, namely the OM signal system (Figure 5b), which only needs a USB cable for data transmission from the acquisition module to the computer. All OM garment contains integrated silicon-based sensors, which can be contacted with skin, machine washable for long-term use (up to several years) and extremely low-cost, so it is favored by the market. Subtle physiological phenomena, such as respiratory sinus arrhythmias, can be easily detected with this new recording system. Another three lead ECG vest using Bluetooth communication is proposed by Liang-Hung et al. [18], which is dedicated to low power design. The bottom of the silica gel dry electrode is in direct contact with human skin; the top is in contact with the conductive button. However, the chest band acquisition system of ZigBee communication powered by battery proposed by Spanò. et al. [71] is 2.4 times lower than its current consumption and 20.72 mW lower than its power consumption, and has a longer life.

Moreover, a variety of smart watch ECG monitoring devices have appeared on the market. They need to be worn for a long time and used in homecare. Among the physiological signal detection products, the sports bra from MoveSense can better fit consumers’ needs, which contains textile electrodes, a detachable Holter containing HR, IMU, and Bluetooth [72]. Alternatively, the MyHeart Instrumented Shirt is equipped with sensors made of conductive and piezoresistive materials, which can monitor ECG, arm EMG, respiratory frequency, skin temperature, and body motion sensors without wireless modules. The whole system only needs a centralized on-body power supply, thus significantly reducing the size of the whole system [73]. After all, the products put on the market are constrained by multiple factors, including battery life, miniaturization, and convenience, as well as price, and even the pursuit of cleanness, practicality, and fashion. Only by continuously optimizing the performance of wearable products and better serving consumers can we truly improve health care. In the above articles, we found that wearable parts are mainly concentrated in the wrists and arms, as well as the chest of upper limbs. Vavrinsky et al. [74] also found that, when measuring ECG and derivative respiration, it is better to use the position under the chest muscle, if possible, the electrode direction is parallel to the heart axis.

In other applications for wearable signs monitoring, we know that ECG monitoring is also very useful for pregnant women and newborns. In order to meet the requirements of long-term continuous fetal monitoring, Chen et al. [19] developed a textile-based dry electrode to measure the ECG signal of infants. The ECG sensor is connected with the acquisition module through five snaps. The acquisition module can save or process the ECG signal and analyze it in real time or offline (Figure 5c). One of the main problems of collecting ECG signals with textile electrodes is the heterogeneity of skin electrode impedance and motion artifacts. Motion artifact refers to the slight displacement between the fabric electrode and the skin caused by the movement, which will lead to a certain offset potential between the electrodes. If there is no buffer, it will cause a serious drift of ECG signals. Chung et al. [75] developed a portable non-invasive sensor, namely, the essential electronic system (EES), which only needs water to attach the sensor to the skin, so as to monitor the vital signs without restriction. The absence of cables makes it easier to handle the infants. Li et al. [76] studied a kind of perinatal pregnant women’s abdominal care belt, combined with the real-time display of mobile phone software, not only realized the remote fetal monitoring but alleviated the problems of pregnant women’s back pain, muscle fatigue, etc. Li et al. [77] developed a single-channel wearable fetal heart sounds measurement device based on an ARM Cortex-M3 microprocessor. Zhu et al. [78] proposed a bi-directional monitoring management mode between pregnant women and obstetrics in the hospital. Through wearable hardware equipment, the data of fetal heart rate was collected and then transmitted to the hospital to realize the remote monitoring of fetal heart rate.

#### 2.3.3. Chemical–Electrophysiological Hybrid Biosensing System

**Figure 6:**
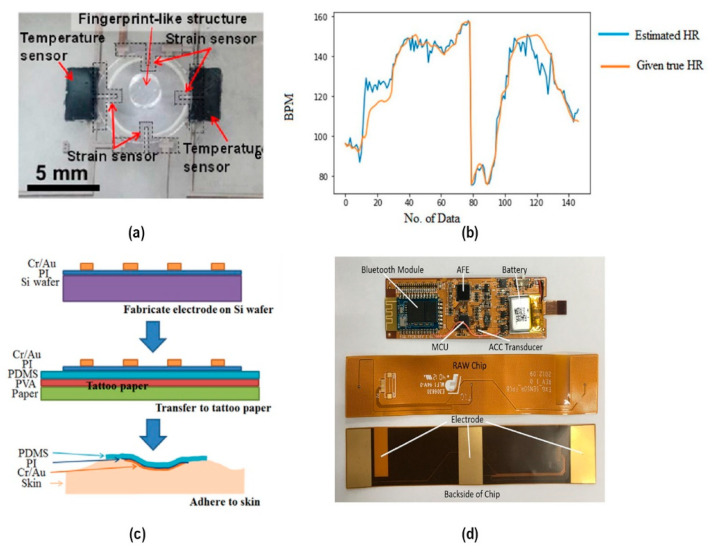
System improvement: (**a**) zoomed-up image of a pixel integrated with four strain sensors, two temperature sensors, and a fingerprint like structure © 2019 IEEE; (**b**) comparison between estimated and given true heart rate for noisy data for matched case using random forest regression with all model features [82] © 2019 IEEE; (**c**) the whole process of handling the flexible and stretchable electrodes [64] © 2014 IEEE; (**d**) wearable ECG monitoring device.

At present, wearable flexible electronic devices are mainly used to test physical signals, such as motion, stress, temperature, etc., which are all biophysical. Since the detection of body chemistry is not good enough, biochemical sensors are still a huge field waiting for development. Imani, S. et al. [79] introduced a skin-worn wearable hybrid sensing system that offers simultaneous real-time monitoring of a biochemical (lactate) and an electrophysiological signal (electrocardiogram), comprising a three-electrode amperometric lactate biosensor and a bipolar electrocardiogram sensor, which can test human health more comprehensively. In recent years, tremendous progress has been made in flexible electronic skin (commonly referred to as “e-skin”). To realize scalability and multifunctionality, especially adding friction force detection, Takei, K. et al. [80] proposed multilayer structures with a suspension sheet, strain sensor sheet, 3-D finger printlike structure sheet, and temperature sensor sheet, and each pixel has four strain sensors, one or two temperature sensors, and a fingerprint like structure (Figure 6a).

For the practical application of this e-skin concept, many issues such as integrating more sensing modalities, improving device reliability, reducing motion artifacts, and interaction between human and e-skin through artificial intelligence and deep learning still need to be addressed in the future. The multi-mode system is one of the most important parts in the future development.

### 2.4. Challenge and Improvement

As illustrated above, motion artifacts seriously affect the quality of signal monitoring. Therefore, it is high time for us to find solutions. First, we should focus on the research of sensing parts, which are expected to eliminate or suppress motion artifacts from the source, such as materials, structure, wearing position, analog front-end and signal conditioning circuit, etc., which have achieved some results. For example, Luo et al. [64] designed a new type of dry electrodes that are light and adhere well to biological tissues, which need both high stretchability and conductivity (Figure 6c). Conventional materials mean low conductivity, while good metal conductors have strong metallic bonds. The author chose to fabricate a thin conductor layer on a flexible substrate such as polydimethylsiloxane (PDMS), including fabricating the serpentine-shape electrode on PI-coated rigid Si wafer, transfer to flexible PDMS-coated tattoo paper by elastic stamp, and adhere to the skin by washing away the tattoo paper.

Second, focus on the software signal process and then use adaptive cancellation algorithms to reduce motion artifacts, which is a major topic of discussion recently. By analyzing the spectral energy changes during the input process of motion artifacts, Xuong et al. [81] found that the motion process exhibits a low-pass nature, that is, most of its spectral energy is concentrated in the low-frequency part, and the process spectrum energy fluctuation increases as the degree of motion of the subject increases. Therefore, a discrete cosine transform-least mean square (LMS) adaptive cancellation algorithm (DCT-LMS) implementation is proposed, aiming to remove the motion artifacts. By collecting ECG signals from fabric-based chest straps with dry electrodes, it is proved that the cosine-based adaptive algorithm behaves better in eliminating high-amplitude motion artifact noise. Another new approach to estimate heart rate without motion effects is called the multi-model machine learning approach (MMMLA) applied by Bashar et al. [82]. It works like this: firstly it trains and tests the model for the different feature and different data set, then separates noisy and non-noisy data by K-means clustering, which lets the machine learn data separately (Figure 6b). This algorithm is used to fit data and predict HR from test data. For ECG detection, Zou et al. [83] proposed a QRS (A pattern seen in an electrocardiogram that indicates the pulses in a heart beat and their duration.) detection-based motion artifact removal algorithm (QRSMR) (Figure 6d). It detects the entire QRS complex and removes the noise between two QRS complexes, while recovering P and T-waves. The QRSMR method can greatly reduce the running time and memory storage, which makes it suitable to implement on wearable ECG monitoring platforms.

## 3. Respiratory Rate Monitoring

With the improvement of human living standards, more and more people are very concerned about their health status, and vital signs can reflect people’s health status, so it is particularly necessary to obtain vital signs. As an important aspect of vital signs, breathing frequency detection and analysis can help people understand their own health to a certain extent. This portion mainly summarizes the research progress of the measurement sensors from the perspective of respiratory sensing, and puts forward the development trends of these aspects in the future.

**Figure 7:**
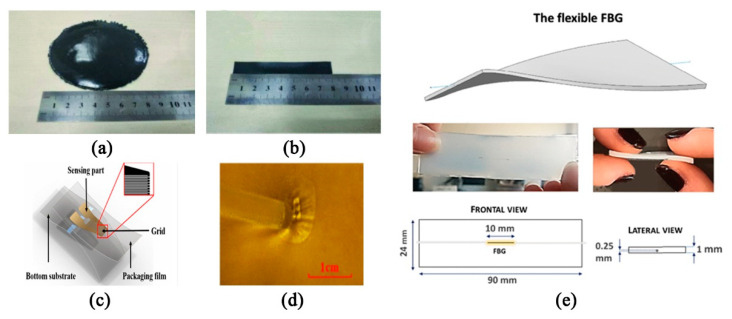
Respiratory monitoring sensors: (**a**) PDMS-graphene compound prototype after the manufacture process and (**b**) a tensile sensor after cutting [86] © 2019 IEEE; (**c**) schematic diagram of a flexible strain sensor with the inset showing the grid part and (**d**) a sensor attached to the skin [93] © 2016 IEEE; (**e**) the flexible fiber-based Bragg grating (FBG) proposed in this work [98] © 2019 IEEE.

Currently, available techniques for detecting breathing frequency mainly include the chest impedance method, three-dimensional acceleration-derived breathing frequency, plethysmography using piezoelectric sensors and breathing induction plethysmography [84,85,86]. At the beginning, chest impedance detection was mostly used, but this method uses electrodes on the skin to obtain signals, and these patches attached to the skin for a long time may cause irritation, so piezoelectric sensors or acceleration sensors are also used to detect the breathing rate. An accelerometer placed on the torso can measure the change in tilt angle during breathing, and then measure the helix rate. Hong et al. [87] adopted a new method based on a chest biaxial accelerometer to derive the breathing rate in static activities. Bates et al. [88] proposed a three-dimensional acceleration or obtaining the respiratory rate from a wireless sensor device, by tracking the rotation axis to obtain a regular rate of respiratory motion. Of course, some scholars have also studied the feasibility of these sensors in respiratory monitoring systems, and found that the respiratory signals collected by piezoelectric sensors or three-dimensional acceleration sensors contain a lot of motion artifacts, which will affect the accuracy of respiratory frequency measurement [86,89]. In order to make the detection method more effective, some works [90,91] focused on the wearable respiratory monitoring device based on respiratory inductance plethysmography (RIP) and used it for respiratory biofeedback training. However, since the device is difficult to embed in a wearable device, it is difficult and complicated to use in practice. In view of this issue, Chen et al. [86] proposed a wearable respiratory monitoring system based on RIP, which uses a new type of PDMS-graphene composite tensile sensor (Figure 7a,b). Compared with traditional RIP-based sensors, the sensors they proposed are more convenient and suitable for wearable products and can be directly combined with clothing as elastic bands. Moreover, the sensor showed high sensitivity during stretching, has good circulation stability in 3600 consecutive cycles and can obtain respiratory signals without interference with high accuracy and satisfactory user experience. Therefore, it has great potential for home monitoring.

Further works are done to optimizing the flexibility and portability for wearable products. Fan et al. [92] proposed a friction electric full textile sensor array with high pressure sensitivity and comfort. The fabricated triboelectric all-textile sensor array (TATSA) can be directly integrated into different parts of the fabric. These parts correspond to the pulse wave at the neck, wrist, fingertip, and ankle, as well as the abdomen and chest breathing waves. This study provides a comfortable, effective, and user-friendly method for measuring human pulse and respiration. In order to meet the biocompatibility of the monitoring device as much as possible, Chen et al. [93] proposed to attach biocompatible and ultra-flexible inorganic strain sensors to the skin to monitor vital signs (mainly pulse and respiratory rate) for a long time (Figure 7c,d). Considering that there are few measurements of breathing rate during exercise, Yamamoto et al. [94] developed a non-invasive strain sensor providing real-time measurements of respiration during exercise. The precision accuracy of the experimental results is high, which also indicates that it may be a useful clinical practice. In addition, fiber optic sensors can be used in any environment because they are not affected by electromagnetic interference, and have received the attention of some scholars in recent years. Kawamura, M. et al. [95] proposed a new vital sign sensing method in 2011, which uses fiber Bragg grating sensors to simultaneously monitor pulse rate and respiration rate. Dziuda, Ł. et al. [96] proposed to use optical strain sensors based on fiber Bragg gratings to monitor the patient’s breathing and cardiac activity during magnetic resonance imaging (MRI) investigations in 2007. Fajkus, M. et al. [97] describes an original simple, low-cost MR fully compatible and safe fiber-optic breathing sensor (FOBS), which can be used for respiratory triggering and monitoring the respiratory frequency in the MR environment. In addition to this, there is a growing demand for strain sensors that can be embedded in wearable devices for a variety of potential applications. Breathing and heart rate monitoring focused on chest wall displacement have promoted the development of strain sensors based on fiber-based Bragg grating (FBG) combined with polymer [98]. The geometric characteristics of flexible fiber gratings and some images related to the sensing elements are shown below (Figure 7e). Of course, the detection accuracy of this optical fiber-based strain sensor needs further experimental evaluation, but it is not difficult to see that the optical fiber-based sensor detection device is a trend for future development.

## 4. Temperature Monitoring

Body temperature can largely reflect people’s physical condition. The body temperature of normal people is relatively constant, which is maintained between 36–37 °C and has nothing to do with the surrounding environment. Normal body temperature is a necessary condition to ensure normal metabolism and life activities. Therefore, in order to monitor body temperature in real time, a variety of flexible temperature sensors have been developed. Generally speaking, there are two ways to measure body temperature, thermistor, and thermoelectric effect.

**Figure 8:**
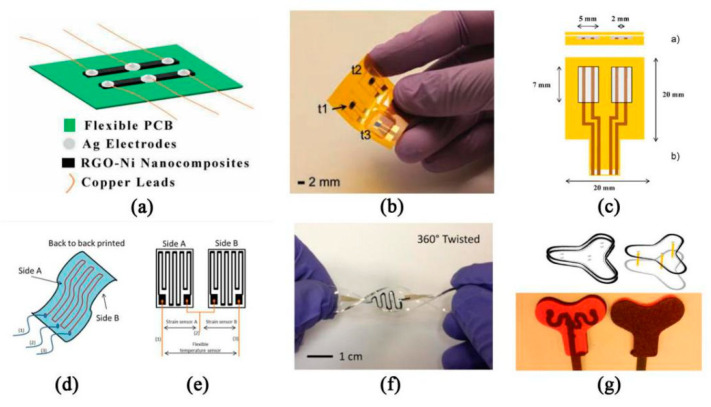
Thermistor temperature sensors: (**a**) schematic diagram of graphene–nickel nanocomposite temperature sensor on a flexible PCB [100] © 2017 IEEE; (**b**) picture of a screen-printed thermistor composed of nickel oxide (NiO) nanoparticles and PSBR adhesive on a Kapton-PI substrate. The four thermistors were labeled t1, t2, t3 and t4, respectively [101]; (**c**) profile and top view of the sensor [102]; (**d**) DTS layout diagram and (**e**) DTS connection diagram [104] © 2019 IEEE; (**f**) actual image of the stretchable graphene thermistor [105].; (**g**) the design structure of the Y-shaped sensor, showing the location of the three pairs of thermistors and the usage of the three heat flows [107] © 2018 IEEE.

The thermistor temperature sensor can be divided into positive temperature coefficient thermistor (PTC) and negative temperature coefficient thermistor (NTC) [99]. They show different resistance values at different temperatures. The higher the temperature is, the higher the resistance of PTC is, and the lower the resistance of NTC is. The temperature coefficient of resistance (TCR) is an important index of sensitivity of resistance temperature sensor, which indicates the relative change of resistance value when the temperature changes 1 °C. Kedambaimoole, V. et al. [100] printed Graphene-Nickel (Ni) nanocomposite film on flexible PCB by screen printing, which is a new method to fabricate temperature sensor arrays. The calculated sensitivity response of the sensor is about 2.455 Ω/K, with TCR about −2.635 × 10^−3^ Ω/K. The sensor manufacturing process is simple, can be mass-produced on PCB, and easy to integrate with electronic devices (Figure 8a). Khan et al. [101] printed NiO/PSBR composite on top of gold electrodes by stencil printing and obtained a high sensitivity thermistor with a temperature coefficient of about −5.84% K^−1^ and a material constant of about 4330 K (Figure 8b). Giuliani et al. [102] adopted a new polystyrene-based ionomer/Multiwalled carbon nanotubes (MWCNT) nanocomposite, which can be made into a small and highly repeatable temperature sensor in the range of 20–40 °C at a low cost (Figure 8c). Wang et al. [103] impregnated the graphene dispersed by sodium alginate solution into the sodium alginate matrix in the dip-coating method, and prepared a flexible temperature sensor (FTS) suitable for human skin, which has good repeatability and stability. In addition, it can eliminate the interference of strain and humidity and distinguish the subtle difference in temperature. In order to further make the flexible temperature sensor easier to stretch, so that the resistance will not produce large error due to the movement of the body, Ali et al. [104] proposed a novel differential temperature sensor (DTS) made of the ink-jet material printer (Figure 8d,e). Compared with the simple resistance temperature detector (RTD), the sensor can measure the temperature of a flexible or curved surface with minimum error. The sensor is ideal for measuring the temperature of a flexible substrate. Yan et al. [105] fabricated a stretched graphene thermistor with high tensile properties by photolithography filtration (Figure 8f). They embed the detection channel and electrode into the elastomer matrix completely, then obtain the equipment that can maintain the function even in the state of high tension. This provides a broad prospect for the application of a wearable temperature sensor.

Most of these studies are based on temperature measurements of the surface of the human skin, but sometimes we need to know the core temperature of the human body. Core body temperature (CBT) refers to the internal temperature of the human body, such as the working temperature of liver, brain, and heart. However, previous measurement methods include direct measurement with esophageal and rectal probes or estimation with mercury and infrared sensors [106]. These measurements are not suitable for all patients, and are not continuous, with errors. In order to estimate the core temperature continuously, Atallah et al. [107] proposed a foam-based Y-shaped sensor with flexible electronic components. The prototype of the sensor is composed of a foam layer, a closed-cell polyethylene foam insulation layer, and thermistor (Figure 8g). With 7.7 min as the average heating time, 0.10 °C for an average error of forehead, 6.9 min as the average heating time of mastoid area, and 0.03 °C as the average error, it can be said to be very suitable for hospital application.

**Figure 9:**
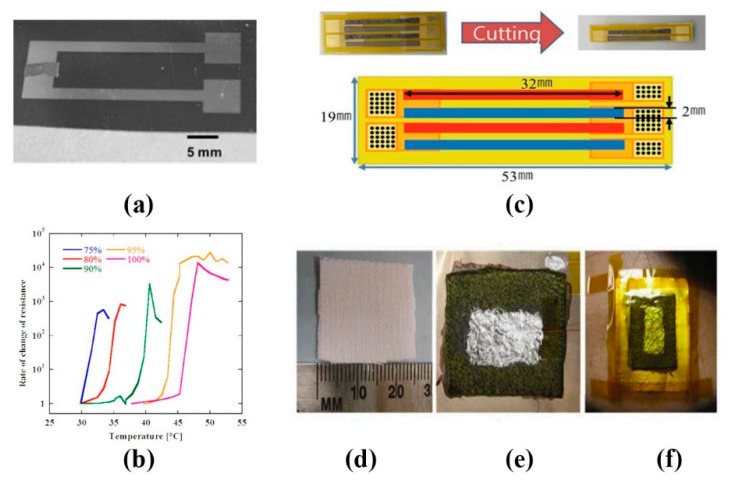
Thermoelectric sensors: (**a**) polymer positive temperature coefficient (PTC) sensor; (**b**) the picture shows the variation of resistance temperature characteristics of the single polymer PTC sensor. The reaction temperature of the sensor varies with the content of octadecyl acrylate from 75% to 100% [111] © 2016 IEEE; (**c**) the size of the flexible TE device [109]; (**d**) fixed-area fabrics; (**e**) devices fabricated on flexible Kapton substrate and (**f**) the device attached to the human body [110].

Thermoelectric sensors can convert temperature changes into changes in resistance, permeability or electromotive force. These changes in electrical parameters can be expressed as temperature variations through appropriate measurement circuits. Tien et al. [108] fabricated flexible pyroelectric OFET devices with piezo-and-pyroelectric nanocomposite gate dielectrics formed by a mixture of P(VDF-TrFE) and BaTiO_3_ nanoparticles, which can effectively distinguish the temperature effect and strain effect of the sensor. Using the co-evaporation method, Lee et al. [109] fabricated thermoelectric (TE) films of n-type bismuth telluride and p-type antimony telluride on PCB (Figure 9c). The sensitivity of the flexible TE temperature sensor was up to 192.70 μVK^−1^. Yang et al. [110] developed a flexible thermoelectric nanogenerator (TENG) based on TE-nanowire/poly (3-hexyl thiophene) (P3HT) polymer composite as a thermoelectric material (Figure 9d–f). The sensor can be self-powered with body temperature as the energy source, with response time of 17 s, reset time of 9 s, and detection sensitivity of 0.15 k. Tatsuya Nakamura et al. [111] proposed a thin, flexible, polymer PTC sensor with a wide temperature measurement range (25–45 °C). They discussed the characteristics of this new type of sensor from three aspects: the characteristics of single polymer PTC sensor, the characteristics of multi polymer PTC sensor and the thickness characteristic (Figure 9a,b). Compared with the traditional polymer PTC, this polymer PTC is more suitable for wearable temperature sensors and medical devices, and its wide temperature measurement range can also help people measure the temperature of various parts of the body in different environments.

## 5. Conclusions

Wearable health monitoring is a rapidly developing field in clinical application, which can improve medical quality and availability. New materials play an important role in solving technical problems, such as high power consumption, low precision, poor reliability and repeatability. This paper reviews the new technologies applied in vital signs monitoring in recent years, especially the monitoring of flexible electrophysiological signals, including ECG, EEG, EMG, etc., and introduces the research progress in respiration and body temperature. With the further development of research and the wider application of sensors, the functions of devices tend from single vital signs monitoring to multiple vital signs monitoring; the design of devices tends to be smaller, lighter, lower cost and have a more comfortable patch design. We have discussed and compared these designs. This review summarizes the monitoring of electrophysiological signals through comparison: the main challenge of the contact sensing method lies in the signal quality and wearing comfort after long-term contact; the main challenge of non-contact sensing method lies in how to effectively overcome motion artifacts; the main challenge of wearable monitoring system lies in its power consumption control as wearable equipment issues, etc. In summary, we have outlined some substantial advances for key vital sign signals in this work. Many other methods and applications will be born and evolved in the future, and their number will continue to grow over time, with bright prospects in diagnosis, treatment and public service.

## Figures and Tables

**Table 1:** Comparation for flexible sensors for vital sign monitoring.

	Vital Signs	Materials	Key Features	Limitations	Reference
Contact sensor	ECG/EMG	Ag NW/PDMS	Anti-microbial, Eliminated motion artifacts	Material oxidation	[6]
	ECG	Polymer/Ag NWs electrode	Highly stretchable, low sensing limit, and good durability	Requires tight contact	[7]
	ECG	Graphene, textile	Easy to make	High noise	[8]
	ECG	PEDOT: PSS, LIG	Prolonged stability, High waveform quality	Prone to motion artifact	[9]
	ECG/EMG	PDMS	Scalable, less skin irritation	Prone to motion artifact	[3]
	ECG	Ag/AgCl	Wi-Fi wireless transmission	High power consumption, short lifespan	[10]
	ECG	Ag/AgCl	Low power consumption, dry 3D printed electrodes	Short battery lifespan	[11]
	EMG	Ag, nylon plastic	Convenient, real time processed	Data accuracy	[12]
Non-contact sensor	ECG/EMG/EEG	PS25255 EPIC	Portability, long-term monitoring	Poor tight contact, prone to motion	[13]
	ECG/EMG/EEG	Flexible printed circuits (FPC)	Flexible, no obvious power frequency noise	Baseline drift exists	[14,15]
	ECG	ASOPA4002	Completely flexible and ultra-thin	High power consumption	[16]
	ECG	Silicone-based sensors	Comfortable, noise immunization	Short monitoring period	[17]
	ECG	Silicone dry electrode	Reliable, low power consumption, low cost,	Irregular waveforms, low CR	[18]
	ECG	PDMS-Graphene	Textile based, high quality	Limited stability	[19]
	ECG	Graphene	Soft, low cost, scalable	Contact impedance exists	[20]
Implant-able sensor	Peripheral neural signals	TiO2, silicone	Good biocompatibility	Unknown mechanical properties	[21]
	ECG	PI, AU/Ti	Flexible, robust performance	High impedance	[22]
	ECG	Ag/AgCl	Low noise, good biocompatibility	High power consumption	[23]
